# Editorial for: Microbial symbiosis of marine sessile hosts- diversity and function

**DOI:** 10.3389/fmicb.2015.00585

**Published:** 2015-06-16

**Authors:** Suhelen Egan, Torsten Thomas

**Affiliations:** School of Biotechnology and Biomolecular Science and Centre for Marine Bio-Innovation, The University of New South WalesSydney, NSW, Australia

**Keywords:** microbial interactions, microbial diversity, sponges, seaweeds, macroalgae, oysters, marine diseases, beneficial microorganisms

The marine surface environment is home to a large and often diverse community of microorganisms. Yet compared to terrestrial ecosystems we still know little about the diversity, degree of host-specificity, functional role or the molecular mechanisms of host-microbe interactions in marine systems. This research topic brings together 10 articles that highlight advances in our understanding of microbial communities associated with marine sessile eukaryotic hosts.

Many papers in this research topic have a particular focus on the stability and diversity of bacterial symbionts of marine sponges. Sponges are a diverse group of sessile organisms, which represent one of the earliest metazoan life forms and play an important role in benthic ecosystems due to their filter feeding activity (e.g., De Goeij et al., 2013). Many biogeochemical processes are carried out by sponge-associated microorganisms, which can comprise up to 35% of the sponge biomass (Hentschel et al., 2012). The evolutionary history and biological importance of the sponge-microbe interaction make them attractive models to study general concepts in marine microbial-host symbiosis. Three articles (Burgsdorf et al., 2014; Cuvelier et al., 2014; Easson and Thacker, 2014) use molecular approaches to elucidate the major factors that determine the composition of sponge symbiotic microbial communities. Burgsdorf et al. (2014) show that the local environment, rather than the host features, influences the community composition of distinct morphotypes of the sponge *Petrosia ficiformis*. In contrast, Cuvelier et al. (2014) conclude for the sponge *Cinachyrella* that the sponge host itself has the greatest influence on determining its microbial community composition. These seemingly opposing views are in part reconciled in the findings of Easson and Thacker (2014) that support the concept of a “core” microbial community in sponges, in line with previous studies (Schmitt et al., 2012), but also highlight that for individual sponge species the taxonomic identity of microbial symbionts can vary greatly.

Using seaweed as another marine model, Campbell et al. (2015) performed a local transplantation experiment to show that the symbiont community of the brown macroalgae *Phyllospora comosa* is primarily influenced by the local conditions, with some evidence for host-specificity. Thus, like sponges, the microbial community composition of seaweeds is also likely determined by a combination of environmental and host factors, a pattern that is emerging now of several sessile marine systems (Wahl et al., 2012; Egan et al., 2013).

Aside from diversity patterns, functional processes such as nitrogen fixation are also important for the dynamics of host-microbe symbiosis, as demonstrated in the research article by Zhang et al. (2014). This study found that expression of nitrogen fixation genes (*nifH*) occurred in two Caribbean sponges over the entire day-night cycle. Comparison between the two sponge species suggested that nitrogen fixation is dominated by a conserved group of cyanobacteria, with the heterotrophic bacterial community mainly contributing during the night.

In contrast to these beneficial aspects of symbiosis, interactions can also be negative and thus result in disease (Webster, 2007; Burge et al., 2013; Egan et al., 2014). Two papers in this research topic examine negative interactions in different marine sessile hosts (Raftos et al., 2014; Zozaya-Valdes et al., 2015). Raftos et al. (2014) review the history and impact of microbial disease on shellfish and using QX disease in Sydney rock oysters, illustrate the complex interactions that exist between pathogens, the environment and hosts. Zozaya-Valdes et al. (2015) provides molecular evidence for the ecological importance of certain bacteria in the bleaching disease of the red macroalga *Delisea pulchra* and also highlight the possibility that multiple opportunistic bacterial pathogens exist.

During the last decade, next-generation sequencing technologies have rapidly advanced our understanding of microbial diversity in the marine environment (Gilbert and Dupont, 2011; Williamson and Yooseph, 2012). However, these culture-independent approaches should be complemented by the culturing of representative microorganisms, followed by detailed physiological, biochemical and genetic studies (Giovannoni and Stingl, 2007; Joint et al., 2010). Hardoim et al. (2014) tackle this challenge using a range of culturing, molecular and microscopy techniques to address cultivation bias when studying microbial communities in sponges. Using a “plate-washing method” they were able to culture an order of magnitude more bacterial species than previous cultivation studies (Hardoim et al., 2012). Approximately half of the bacterial species cultured were not detected in the sponge by culture-independent methods demonstrating the need for these complementary approaches to be used to fully characterize microbial diversity.

Having model bacteria is clearly important to explore mechanistic aspects of symbiosis. The article by Gardiner et al. (2014) illustrates this by showing that the seaweed-associated bacterium *P. tunicata* utilizes a surface lipoprotein (designated Ptl32) to attach to its host. Interestingly, Ptl32 shares homology to a conserved protein in *Leptospira* species, the causative agent of leptospirosis in animals (Murray, 2013), and this suggests that this attachment mechanism might be distributed between distantly related bacterial species via horizontal gene transfer (HGT). A clear link between organisms and their functions is important to define the importance of HGT in marine microbial symbiosis and this is further explored in the final article of this research topic. Here, Degnan (2014) raised a number of compelling points of how HGT between symbiotic microorganisms and marine invertebrates can shape the evolution of holobionts. HGT is clearly an underappreciated mechanism by which bacteria can influence their host (or vice versa).

The articles presented in this research topic highlight the diverse microbial communities associated with marine sessile macroorganisms play important roles in their health, function and evolution. Through the contributions of experts in classical microbiology (Gardiner et al., 2014; Hardoim et al., 2014), cell biology (Raftos et al., 2014), ecology (Campbell et al., 2015), evolution (Degnan, 2014) and molecular ecology (Burgsdorf et al., 2014; Cuvelier et al., 2014; Easson and Thacker, 2014; Zhang et al., 2014; Zozaya-Valdes et al., 2015), the papers in this research topic also demonstrate the benefits of using a multidisciplinary approach to understand the diversity, function and evolution of complex symbiotic systems.

## Conflict of interest statement

The authors declare that the research was conducted in the absence of any commercial or financial relationships that could be construed as a potential conflict of interest.
